# Auxetic Cardiac Patches with Tunable Mechanical and Conductive Properties toward Treating Myocardial Infarction

**DOI:** 10.1002/adfm.201800618

**Published:** 2018-05-24

**Authors:** Michaella Kapnisi, Catherine Mansfield, Camille Marijon, Anne Geraldine Guex, Filippo Perbellini, Ifigeneia Bardi, Eleanor J. Humphrey, Jennifer L. Puetzer, Damia Mawad, Demosthenes C. Koutsogeorgis, Daniel J. Stuckey, Cesare M. Terracciano, Sian E. Harding, Molly M. Stevens

**Affiliations:** Department of Materials, Department of Bioengineering, Institute of Biomedical Engineering, Imperial College London, SW7 2AZ London, UK; National Heart and Lung Institute, Imperial College London, W12 0NN London, UK; Department of Materials, Department of Bioengineering, Institute of Biomedical Engineering, Imperial College London, SW7 2AZ London, UK; National Heart and Lung Institute, Imperial College London, W12 0NN London, UK; National Heart and Lung Institute, Imperial College London, W12 0NN London, UK; Department of Materials, Department of Bioengineering, Institute of Biomedical Engineering, Imperial College London, SW7 2AZ London, UK; School of Science and Technology, Nottingham Trent University, NG11 8NS Nottingham, UK; Centre for Advanced Biomedical Imaging, University College London, WC1E 6DD London, UK; National Heart and Lung Institute, Imperial College London, W12 0NN London, UK; Department of Materials, Department of Bioengineering, Institute of Biomedical Engineering, Imperial College London, SW7 2AZ London, UK

**Keywords:** auxetic materials, biomaterials, cardiac patches, doped-conjugated polymers, re-entrant honeycombs

## Abstract

An auxetic conductive cardiac patch (AuxCP) for the treatment of myocardial infarction (MI) is introduced. The auxetic design gives the patch a negative Poisson’s ratio, providing it with the ability to conform to the demanding mechanics of the heart. The conductivity allows the patch to interface with electroresponsive tissues such as the heart. Excimer laser microablation is used to micropattern a re-entrant honeycomb (bow-tie) design into a chitosan-polyaniline composite. It is shown that the bow-tie design can produce patches with a wide range in mechanical strength and anisotropy, which can be tuned to match native heart tissue. Further, the auxetic patches are conductive and cytocompatible with murine neonatal cardiomyocytes in vitro. Ex vivo studies demonstrate that the auxetic patches have no detrimental effect on the electrophysiology of both healthy and MI rat hearts and conform better to native heart movements than unpatterned patches of the same material. Finally, the AuxCP applied in a rat MI model results in no detrimental effect on cardiac function and negligible fibrotic response after two weeks in vivo. This approach represents a versatile and robust platform for cardiac biomaterial design and could therefore lead to a promising treatment for MI.

## 1 Introduction

Cardiovascular diseases (CVDs) are the leading causes of death and disability worldwide.[1] Biomaterials and regenerative therapies represent exciting possible solutions.[2–8] Herein, we investigate a new biomaterial design for cardiac patches to treat myocardial infarction (MI), one of the largest contributors to CVDs. MI are caused by an occlusion of one or more of the coronary arteries, resulting in the myocardial tissue becoming ischemic and reducing the heart’s ability to pump blood around the body.[9] Cardiac patches seek to strengthen the tissue, supply cells or growth factors to vitalize the tissue, and bridge electrical and/or mechanical stimulation across the infarct to maintain and improve cardiac function. For a cardiac patch to be successful in triggering regeneration of the myocardium, the biomaterial design will benefit from considering and optimizing the cytocompatibility, electrical conductivity, and mechanical properties.[2–8]

It is widely accepted that the mechanical properties of biomaterials used for treating MI are extremely important.[2–8,10] Typically, it is believed that the mechanical properties of a cardiac patch should match those of healthy native heart tissue. However, the Young’s modulus of the native human heart varies from 0.02 to 0.50 MPa depending on whether the heart is in systole or diastole, with infarct tissue being even stiffer.[7,8] This range is broad and the optimum mechanical properties for such an application are debatable.[7,8] Further research into the regeneration of infarcted cardiac tissue using cardiac patches with controllable mechanical properties will be instrumental in the development of future strategies for treating MI.[7,8]

Another important mechanical property which is often overlooked is the Poisson’s ratio. The Poisson’s ratio (*ν*) describes the effect on transversal strain (*ε*_T_) when a material is under longitudinal tension or compression, creating longitudinal strain (*ε*_L_), and is defined by Equation (1)

(1) $$\nu =-\frac{\varepsilon_{\text{T}}}{\varepsilon_{\text{L}}}$$

Most materials typically have a positive Poisson’s ratio and as a result, when stretched longitudinally, they contract transversally. However, auxetic materials are defined as having a negative Poisson’s ratio and therefore expand in multiple directions simultaneously. This results in the improvement of other unique properties, such as shear resistance, indentation resistance, and synclastic curvature, all of which are interesting properties in a cardiac patch.[11–13] Auxetic micropatterning is presented here as a unique way of incorporating these properties into cardiac patches. There has been very little investigation into the use of auxetic materials or the importance of the Poisson’s ratio in the field of biomaterials, particularly for treating MI.[14–17]

In addition to mechanical strength, electrical signals are vital to the heart’s contractions and function. Due to their inherent electroactive nature, conductive materials are of interest in biomaterial design for electroresponsive tissues such as the heart and the central nervous system. Doped-conjugated polymers could be particularly useful in cardiac patches due to their potential ability to improve the electrical pathway which is damaged in the infarct tissue after an MI.[18–23] Electroactive biomaterials were recently shown to favorably influence the electrophysiology of infarcted animal cardiac tissue ex vivo[24] and in vivo.[19] In contrast, the existing insulating biomaterials have been shown to further hinder signal propagation through the infarct tissue.[25]

Previously, our group developed a conductive cardiac patch with exceptional electrical stability, which was shown to increase the conduction velocity (CV) across the damaged electrical pathway of the infarct region in a rat MI model.[24] However, the Young’s modulus of this biomaterial is significantly greater (6.73 ± 1.1 MPa)[24] than that reported for native human heart tissues (0.02–0.50 MPa).[7,8] Building on our previous work,[24] here we incorporate mechanical and topological anisotropy in an auxetic design to develop a conductive cardiac patch that can better comply mechanically with the heart. We introduce the ability to control and tune the effective stiffness and anisotropy of auxetic, conductive cardiac patches and we investigate their impact on cardiac function.

## 2 Results and Discussion

### 2.1 Design and Fabrication of Auxetic Cardiac Patches

**Figure 1:**
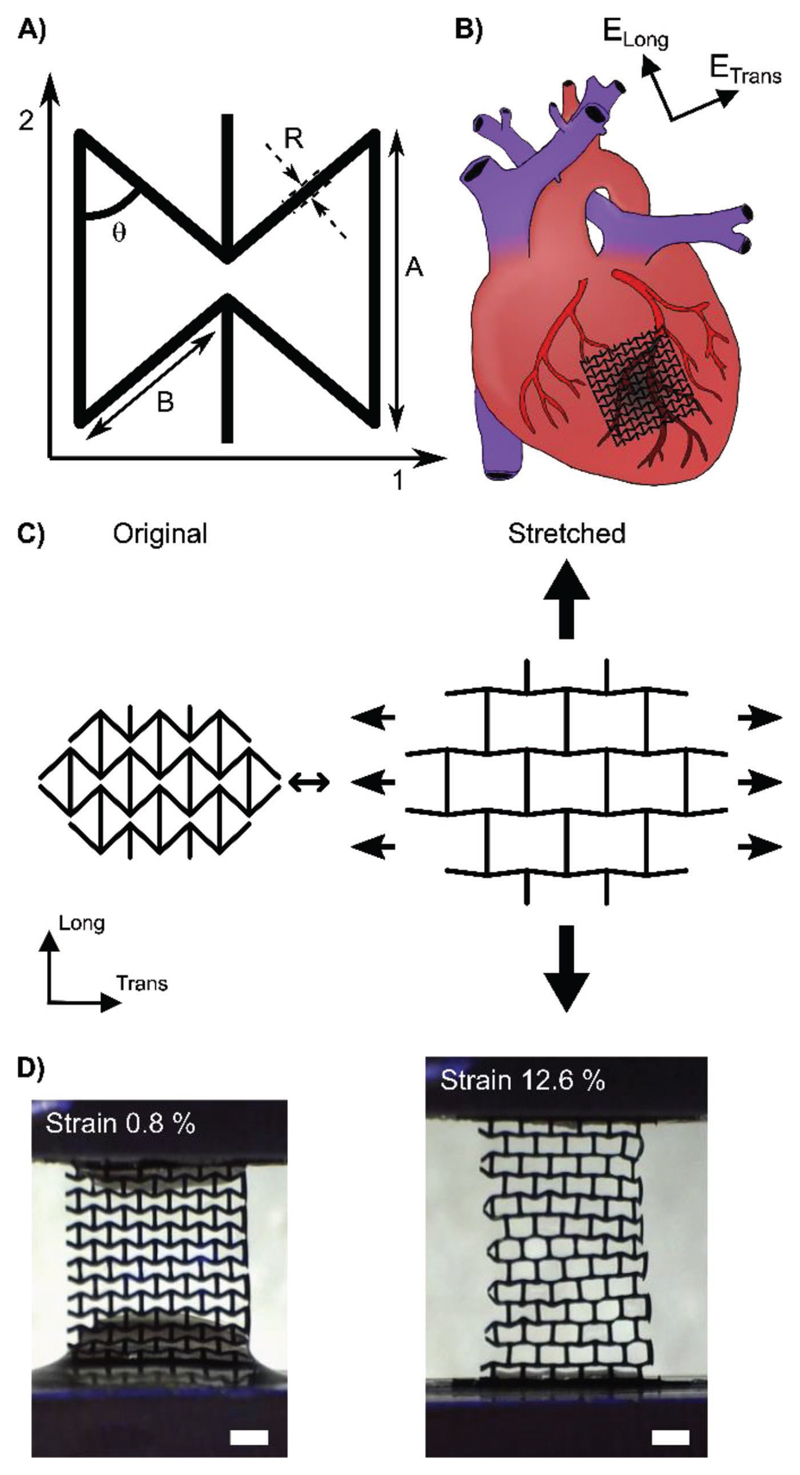
A) Schematic of the bow-tie dimensions. B) Schematic illustration of the alignment of the auxetic cardiac patch (AuxCP) on the heart. C) Schematic illustration of the auxetic behavior of the re-entrant honeycomb (bow-tie) geometry. D) Digital optical microscope images of the AuxCPs during tensile testing at 0.8% strain and 12.6% strain (scale bars: 1 mm).

There are many ways in which auxetic behavior can be imparted into a material.[13] The design used here is known as the re-entrant honeycomb or “bow-tie” deformation mechanism (Figure 1A).[13,26] A particularly interesting feature of this bowtie geometry is that it is anisotropic, that is, it is stiffer in one direction than the other, which is similar to native heart tissue. The anisotropic ratio of effective stiffness of native hearts has been reported over a wide range of values (1.9–3.9),[27–30] depending on species, age, and health. However, it is consistently reported that the heart is stiffer in the circumferential (transverse) direction than in the longitudinal direction.[27–30] It is assumed that cardiac biomaterials should match native anisotropic mechanical properties to not impede heart function.[27–30]

In this study, the stiffer direction (1-direction) of the auxetic cardiac patch (AuxCP) is aligned with the stiffer circumferential (transverse) direction of the heart, while the less stiff AuxCP direction (2-direction) is aligned with the less stiff longitudinal direction of the heart (Figure 1B). When this bow-tie pattern is stretched in one direction, the diagonal ribs flex and open causing expansion in the other direction, resulting in a negative Poisson’s ratio (Figure 1C,D). The Poisson’s ratio is negative within the first 5–15% strain (depending on original bow-tie dimensions), a representative example is illustrated in Figure S1 (Supporting Information).

The material used is a conductive composite previously developed by our group.[24] Briefly, this composite consists of an interconnected network of polyaniline and phytic acid grown on a chitosan surface. Ammonium persulfate is used to trigger the polymerization of the aniline in situ, while the phytic acid cross-links both the chitosan and polyaniline. The phytic acid also has a second role as the dopant, resulting in a highly stable, electrically conductive thin film with the rich dark green color associated to the emeraldine acid oxidation state of polyaniline.

The fabrication of the bow-tie geometry was achieved through excimer laser microablation in the chitosan films. The process can be easily modified to create patches with varying bow-tie geometries and sizes (Figure 1A). After micropatterning the chitosan, the patches are coated with polyaniline and their precision is maintained to within 5% of the original microablated pattern dimensions (Figure S2, Supporting Information). Unlike other micropatterning methods such as photolithography, this technique is not limited to photocurable materials,[7] opening the door to the micropatterning of a wide array of biomaterials, particularly conductive biomaterials. In addition, the fabrication process was optimized to produce 40 samples (6 × 6 bow-tie repeat units) in under an hour while maintaining the precision of the patterning, producing cardiac patches considerably faster than can be achieved with many other rapid prototyping methods, such as common 3D printing techniques.[17] Therefore, this technique has great potential for mass production and distribution. Moreover, this is an extremely robust and versatile technique for 2D microfabrication of biomaterials that allows tissue specific tailoring.

### 2.2 Characterization of Tunable Mechanical Properties

Theoretical models based on the 2D bow-tie pattern, previously established in the literature,[26] were used to predict the mechanical properties of the micropatterned patches. The equations use the Young’s modulus of the bulk material and the various bow-tie dimensions (Figure 1A) to calculate the resultant effective stiffness (*E*) values and the anisotropic ratio of effective stiffness (*E*_1_/*E*_2_).

**Figure 2:**
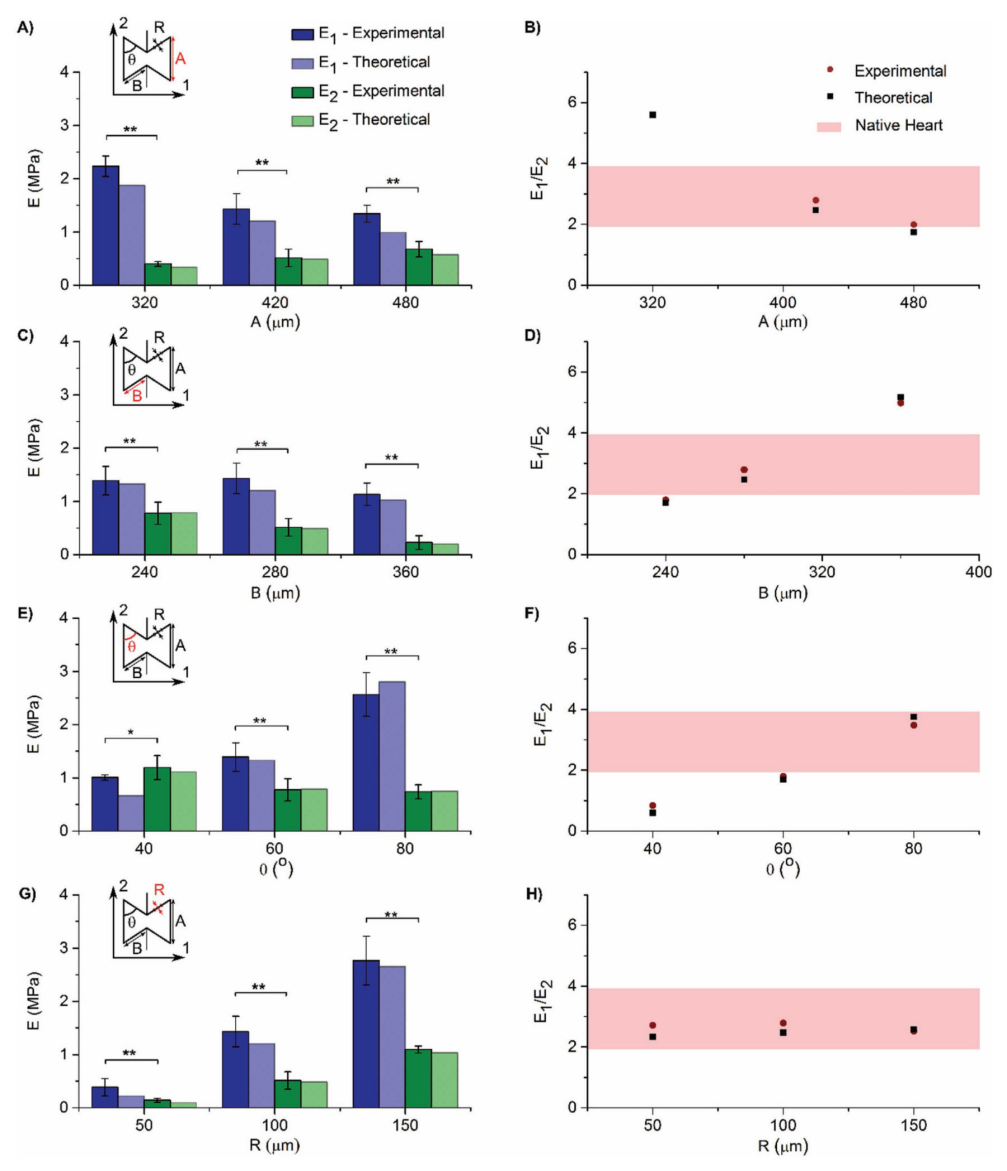
A,C,E,G) Individual effective stiffness (*E*1 and *E*2) compared with theoretical predictions, for changing bow-tie dimensions *A*, *B*, *θ*, and *R*, respectively. B,D,F,H) Anisotropic ratio of effective stiffness (*E*1/*E*2) compared to theoretical predictions and native rat hearts, for changing bowtie dimensions *A*, *B*, *θ*, and *R*, respectively. (Theoretical predictions were calculated from ref. [26]. Adult rat heart tissue values were taken from refs. [27–30]). (*N* = 10, *p < 0.05, **p < 0.001; Mean ± SD).

Tensile tests proved that varying the bow-tie dimensions *A*, *B*, *θ*, and *R*, can tune *E*_1_, *E*_2_, and E_1_/E_2_ as predicted by the theoretical model[26] (Figure 2A–H). A full table of information about all nine different sample sets can be found in Table S1 (Supporting Information), including all bow-tie dimensions and measured mechanical properties. (Representative stress– strain curves produced from these tensile tests are also shown in Figure S3, Supporting Information.)

Specifically, by increasing the length of dimension *A* from 320 to 480 μm, *E*_1_/*E*_2_ decreases from 5.71 to 1.99 (Figure 2A,B). By increasing the length of dimension *B* from 240 to 360 μm, *E*_1_/*E*_2_ increases from 1.79 to 5.11 (Figure 2C,D). By increasing angle *θ* from 40° to 80°, *E*_1_/*E*_2_ increases from 0.85 to 3.48 (Figure 2E,F). Hence, by varying any of these three dimensions, we can tune the cardiac patch to have an anisotropic ratio of effective stiffness matching the reported ratio for native heart tissue (rat heart *E*_trans_/*E*_long_: 1.9 to 3.9).[27–30] This technique provides greater flexibility and control over a wide range of anisotropic mechanical properties, allowing the material to be tailored to match patient needs with varying ages or health conditions. This is in contrast to Engelmayr et al. who achieved a fixed anisotropy of effective stiffness of 2.7 with their nonconductive, nonauxetic micropattered cardiac patch.[29]

Varying *R* has little effect on *E*_1_/*E*_2_ (Figure 2G,H). However, when decreasing *R* from 150 to 50 μm, the magnitudes of *E*_1_ and *E*_2_ decrease from 2.77 ± 0.5 to 0.39 ± 0.2 MPa and from 1.10 ± 0.1 to 0.14 ± 0.04 MPa, respectively, providing an additional level of control in mechanical properties. As well as matching the anisotropic ratio of effective stiffness, this method allows us to match the effective stiffness of the cardiac patch to native human heart tissue (0.02–0.50 MPa).[7,8]

Ultimate tensile strength (UTS) and strain at failure are in the range of 0.06 ± 0.03 to 1.53 ± 0.9 MPa and 27 ± 6 to 96 ± 29%, respectively, which are comparable to reported values for native human heart tissue.[7,31] Poisson’s ratio values were negative for all but one of the sets of bow-tie dimensions. The exception was for the patch with an angle between the ribs of 80°, which leaves little room for it to expand laterally when stretched (Poisson’s ratio of *ν*_12_: 0.00 ± 0.00 and *ν*_21_: 0.08 ± 0.1). The majority of the patches were found to have Poisson’s ratios in the range of −1.45 ± 0.2 to −0.15 ± 0.1 (Table S1, Supporting Information). The Poisson’s ratio can also be tuned to a desired value by tuning the bow-tie dimensions, as has been demonstrated before with a nonconductive poly(ethylene glycol) material by Zhang et al.[15] They developed a biomaterial patterned with the same re-entrant honeycomb geometry with a tuned Poisson’s ratio for the purpose of probing how cells sense and respond to these local subtle differences in mechanical properties.[15] However, their two-photon fabrication technique is restricted to photocurable materials, whereas the excimer laser microablation fabrication techniques used here can be used for almost any biomaterial.

As a result of the wide range of effective stiffness (human heart: 0.02–0.50 MPa)[7,8] and anisotropy (rat heart: 1.9–3.9),[27–30] reported for native healthy heart tissues, the ideal mechanical properties for a cardiac patch are still under debate. Our new approach provides a promising technique to probe the appropriate biomaterial mechanical properties for treating infarcted hearts. The biomaterials can be tuned to a wide range of desired values of effective stiffness and anisotropy while maintaining the bulk properties. Moreover, this control over the mechanical properties is particularly exciting as it can be extended to other tissues and applications where the tissues are expanded under stress, such as arterial stents,[32] esophageal stents,[14] and osteochondral implants[33,34] among others.

### 2.3 Characterization of Conductivity

**Figure 3:**
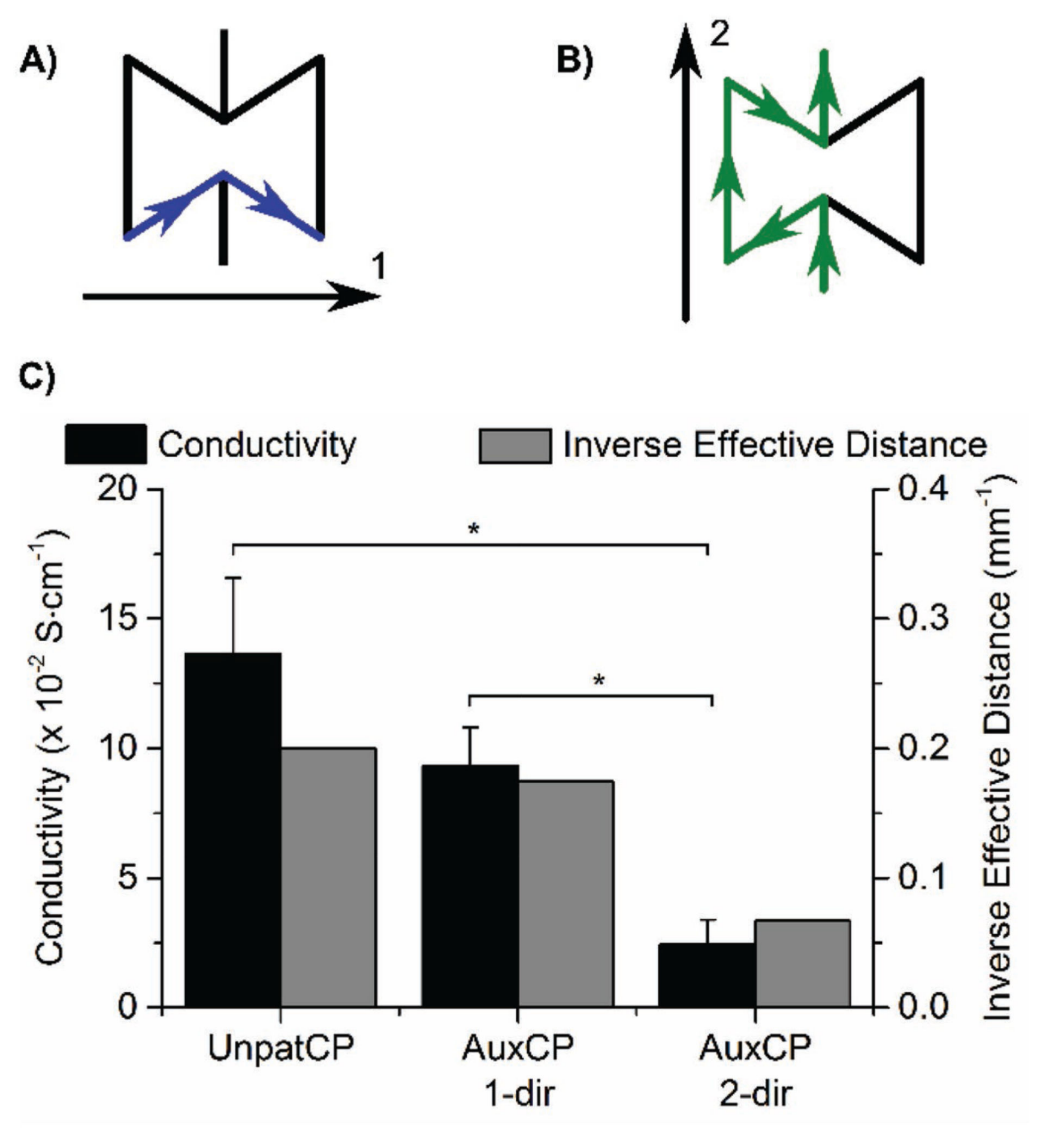
A,B) Schematic of the route the current travels along the patch in the 1- and 2-directions, respectively. C) Two-point probe conductivity measurements for the unpatterned cardiac patches (UnpatCPs) and the auxetic cardiac patches (AuxCPs) in both the 1- and 2-directions, correlated with the inverse effective distance that the current travels along the patch (*N* = 3–5, *p < 0.05; Mean ± SD).

The AuxCPs maintain a similar level of conductivity compared to the unpatterned cardiac patches (UnpatCPs) previously produced,[24] with values in the 10^−2^ S cm^−1^ range (UnpatCP: 13.6 ± 2.9 × 10^−2^ S cm^−1^, AuxCP 1-dir: 9.3 ± 1.5 × 10^−2^ S cm^−1^ and AuxCP 2-dir: 2.4 ± 0.9 × 10^−2^ S cm^−1^; Figure 3). Interestingly, the conductivity of the AuxCP is also anisotropic. The data show that conductivity is significantly higher in the 1-direction than the 2-direction. Similarly, the results also show that the UnpatCP has a significantly higher conductivity than the AuxCP in the 2-direction. The stated conductivity values are calculated by taking the length between the two electrodes as the current travel distance. While this is true for the UnpatCP, it is more complex for the AuxCP. By following the pattern in each direction, it can be seen that the distance the current travels is longer than the distance between the two electrodes, with the longest route being in the 2-direction. The inverse of these calculated effective distances correlates well with the trend in conductivities, as can be seen in Figure 3. Overall the patterning has a significant yet small effect on the conductivity of the patches, as they maintain conductivities in the 10^−2^ S cm^−1^ range.

The conductivity values measured for the AuxCPs are comparable with other polyaniline-based composites used for cardiac tissue engineering from literature. For example, the polyaniline-poly(glycerol sebacate) composite produced by Qazi et al.[35] has conductivities in the 10^−3^ S cm^−1^ range with 15 v/v% polyaniline and in the 10^−2^ S cm^−1^ range with 30 v/v% polyaniline.[35] However, we previously reported that our chitosan-polyaniline composite has superior electrical stability over other such polyaniline-based composites,[24] a vital characteristic for tissue engineering applications. Moreover, the electrical conductivities reported here for the AuxCPs fall within the range believed to be relevant for tissue engineering applications (10^−4^–10 S cm^−1^).[20] However, the ideal electrical conductivity for cardiac biomaterials is still undefined and is an area that would benefit from further investigation.

### 2.4 Neonatal Rat Ventricular Myocytes Cultured on Auxetic Cardiac Patches

In vitro cell culture experiments were carried out to demonstrate the cytocompatibility of the patches, prior to ex vivo and in vivo experimentation. Both neonatal rat ventricular myocytes (NRVM) and fibroblasts were cultured on AuxCPs for 3 d (Figure S4A,B, Supporting Information). The cells were well dispersed along the pattern and presented the elongated phenotype expected of cardiomyocytes. Moreover, the apparent striation pattern, positive for *α*-actinin, confirms cardiomyocyte phenotype (Figure S4B, Supporting Information). Some NRVMs can be seen to take on a more circular morphology and form aggregates, suggesting dead cells. However, a cell proliferation assay (see the Experimental Section, Supporting Information, for a description) demonstrates that cells on both the AuxCPs and UnpatCPs have significantly higher levels of cell metabolic activity compared to the glass control (Figure S4C, Supporting Information). Hence, both the AuxCPs and UnpatCPs can be considered cytocompatible for both cardiac fibroblasts and cardiomyocytes.

### 2.5 Ex Vivo Investigation of Cardiac Electrophysiology

After optimizing the auxetic mechanical properties, characterizing the conductivity and demonstrating the cytocompatibility of these cardiac patches, we investigated the impact of the patches on the electrophysiological and mechanical function of the heart. First, to probe the effect of the AuxCPs electrical conduction, we carried out ex vivo experiments on both ultrathin myocardial slices and whole rat hearts[24] using AuxCP-1 (Table S1, Supporting Information; setup number 1). The results suggested that AuxCP-1 had an anisotropy in conductivity between directions 1 and 2 (Figure 3) and was therefore an interesting candidate for investigating the impact of its electrical conductivity when attached to the heart tissue in two possible orientations (1-dir: where the more conductive 1-direction of the AuxCP is positioned parallel to the longitudinal axis of the cardiac tissue; 2-dir: where the more conductive 1-direction of the AuxCP is positioned perpendicular to the longitudinal axis of the cardiac tissue).

#### 2.5.1 Impact of Auxetic Cardiac Patches on Myocardial Slices

**Figure 4:**
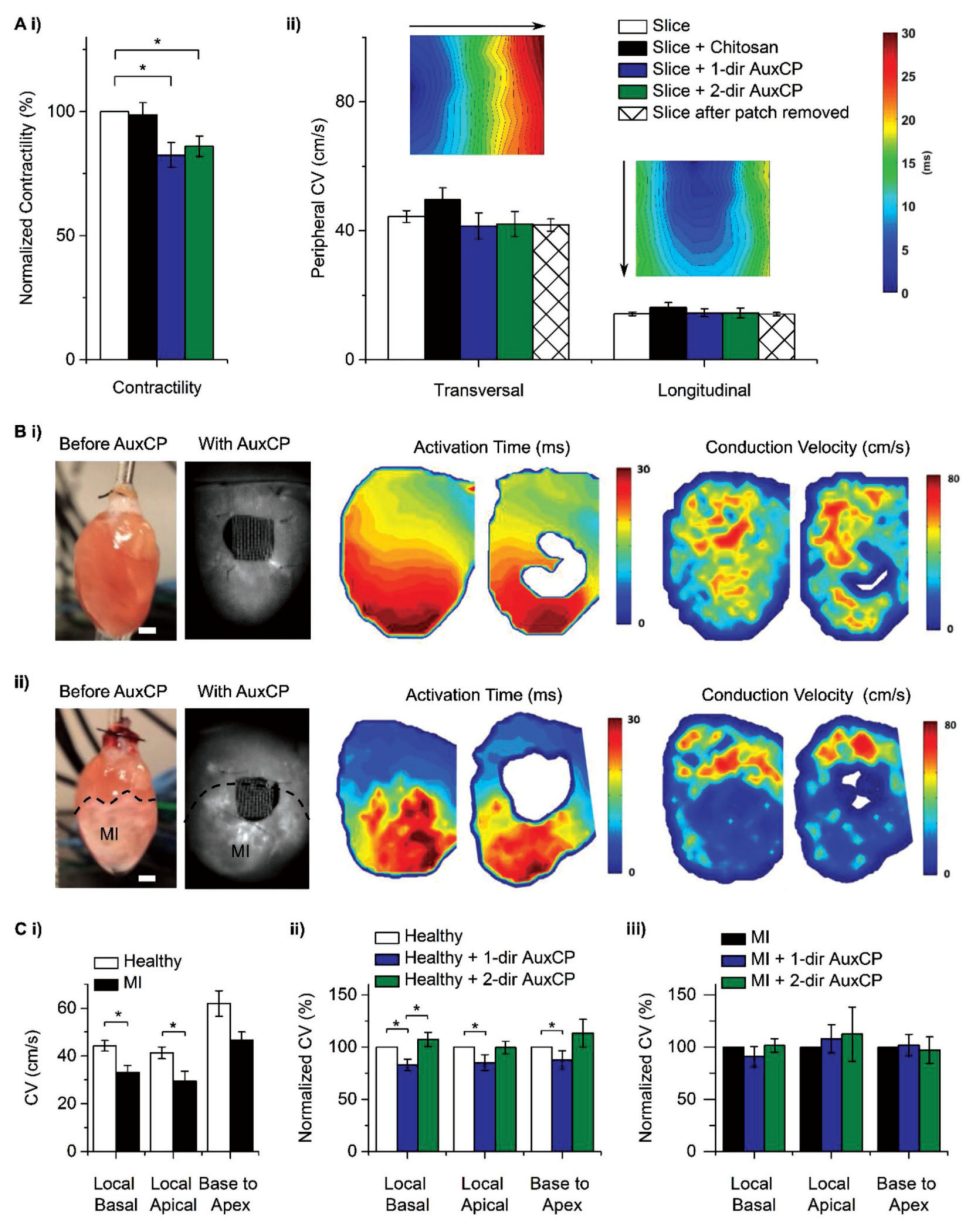
Ai) Contractility of the myocardial slices in response to field stimulation (*N* = 12–16, *p < 0.05; Mean ± SE). Aii) Peripheral conduction velocity (CV) of the slices measured by an MEA system, including CV maps (*N* ≥ 6, *p < 0.05; Mean ± SE). B,C) Ex vivo whole heart optical mapping measurements. B) Photos (scale bar: 2 mm), representative activation time maps and CV maps before and after auxetic cardiac patch (AuxCP) application (1-dir), on Bi) healthy hearts and Bii) hearts two weeks after induced myocardial infarction (MI; permanent ligation of left anterior descending coronary artery). Ci) CV in healthy and MI hearts (*N* ≥ 10, *p < 0.05; Mean ± SE). CV in Cii) healthy hearts and Ciii) MI hearts after patch application, normalized to the hearts without the patch (*N* ≥ 5, *p < 0.05; Mean ± SE).

Upon electrical field stimulation, we observed a reduction in contractility when placing the AuxCPs on myocardial slices, compared to the slices on their own. This is reported as the relative contractility, that is, 100% without a patch to; Slice + 1-dir AuxCP: 82.6 ± 5.0% and Slice + 2-dir AuxCP: 86.0 ± 4.1%; Figure 4Ai. However, we did not observe a significant difference between the two possible AuxCP orientations. In both cases, we observed a reduced contractility of the slices. We hypothesize that this effect is due to the electrical conductivity of the AuxCP. To this end, we conducted the same experiment with nonconductive chitosan patches, consisting of the same auxetic micropattern yet without the polyaniline coating. Contractility upon electrical stimulation of the myocardial slices with this chitosan patch was comparable to slices without any patch. This suggests that the presence of the doped-polyaniline is critical to the observed reduction in contractility, which could be attributed to the electroactive nature of the polyaniline-chitosan composite patch compared to the chitosanonly patch.

This is comparable with our previous work, which found that the UnpatCPs reduced the contractility of the slices while the chitosan-only patches of the same architecture did not influence the contractility of the slices.[24] Interestingly, however, this previous work also showed a size-dependent effect, with the larger (25 mm^2^) UnpatCPs having the greatest effect on contractility compared to the smaller patches (17 and 9 mm^2^), where no reduction in contractility was observed.[24]

In the experiments reported here, we used AuxCPs of similar dimensions (21.1 mm^2^). Taking the patterning into consideration, the total surface area of the AuxCPs, and therefore the material placed on the myocardial slices, is considerably less (10.0 mm^2^). Contrary to our expectations and our previous work, this reduced surface area still affected the contractility of the myocardial slices. We thus propose that the contractility of myocardial slices upon electrical stimulation is influenced not only by the size, weight, and conductivity of a patch, but also by its geometry and auxetic properties. The ex vivo evaluations described here further underpin the importance of considering various aspects in cardiac patch design, and highlight the complexity in trying to elucidate the role of these different parameters from one another experimentally. To gain further insight into the true effect of the AuxCPs on cardiac function, further ex vivo, as well as in vivo experiments have been conducted and their descriptions follow.

A microelectrode array (MEA) system[24] was also used to examine the AuxCPs on the myocardial slices, which measures signal propagation through the tissue upon electrical field stimulation, allowing activation time maps to be produced along with the corresponding calculated peripheral CVs (Figure 4Aii). Because CV is faster along the fibers,[24] the slice is stimulated in two orientations, longitudinal in-line with the fibers and transverse across the fibers. The peripheral CVs for both slice orientations (longitudinal and transverse) are unaffected by the addition of the AuxCPs in either direction (1- or 2-direction) and there is no significant difference seen for the chitosan patches (Figure 4Aii).

Previously, we have reported a reduced CV for the myocardial slices with UnpatCPs both in the transversal and longitudinal directions on the MEA system.[24] Here, we show that the AuxCPs do not restrict the CV neither in the transversal nor in the longitudinal directions on the MEA system, which could be due to the significantly smaller surface area of the AuxCPs (10 mm^2^) compared to the UnpatCPs (25 mm^2^). However, this would contradict the results seen for the force transducer contractility measurements. The MEA system stimulates and reads the response specifically from the bottom side of the tissue while the patch is placed on the top side of the tissue. This is different from the force transducer contractility measurements, which measures an overall effect on the tissue. Hence, it is also possible that the effect of the AuxCPs on CV is restricted to the surface of the tissue and does not pass all the way through the tissue (≈15 cell layers) to the MEA dish electrodes.

#### 2.5.2 Impact of Auxetic Cardiac Patches on Whole Hearts

Optical mapping experiments[24] were conducted to investigate the effect of the AuxCPs on the electrophysiology of whole hearts. The optical mapping measurements were taken before and after application of the AuxCPs. The optical measurements were processed to produce activation time maps and conduction velocity maps (Figure 4B). As expected, prior to application of the patches, the CV is significantly reduced in the MI hearts compared to the healthy hearts (Figure 4Ci). Similar to the heart slice measurements, the patch was placed in two different orientations. The orientation of the patch had an impact on the CV for healthy hearts. The patch oriented in the 1-direction significantly reduced the CV (from 100% without a patch to 82.9 ± 5.5, 84.9 ± 7.7, and 87.5 ± 8.9% for the local basal, local apical, and base to apex measurements, respectively). The patch oriented in the 2-direction had no significant impact on the CV of healthy hearts (Figure 4Cii). In contrast, with MI hearts neither orientation of the patch had a significant impact on CV (Figure 4Ciii).

The results for the UnpatCPs and nonconductive unpatterned chitosan patches were published previously[24] and so for animal welfare reasons these experiments were not repeated. We previously showed a significant decrease in CV in healthy hearts and a significant increase in CV in MI hearts when the UnpatCPs were applied, and no significant difference in either case for the chitosan patches.[24] This whole heart CV data combined with the myocardial slice CV data suggests that the UnpatCPs could influence the electrophysiological signaling of the heart.[24] In this study, we show that the AuxCPs do not induce a significant difference in CV for both healthy and MI whole hearts and healthy slices, when oriented in the correct direction (2-dir: less stiff patch direction aligned with less stiff longitudinal direction of the heart). Furthermore, an additional AuxCP (AuxCP-10) was designed with a new set of bow-tie dimensions to maximize surface area (AuxCP-10 = 18.7 mm^2^, AuxCP-1 = 10.0 mm^2^), and this design did not influence the CV of either healthy or MI hearts in both 1- and 2-directions (Table S2 and Figure S5, Supporting Information). Therefore, this demonstrates that the AuxCPs do not have a detrimental effect on the electrophysiology of both healthy and infarcted hearts over a wide range of dimensions and surface areas.

Nevertheless, these preclinical experiments cannot fully predict the effect of AuxCPs on human heart electrophysiology and function. As auxetic behavior is independent of scale, larger AuxCPs will maintain similar mechanical properties. In addition, the excimer laser microablation process lends itself well to scale-up; with the ability for high-throughput production; and patch size only limited by the XY stage dimensions (currently ≈150 × 150 mm) and thus theoretically suitable for up to human heart dimensions. However, left ventricular wall thickness and heart rate are significantly different between rats and humans, and the effects of patch application in larger animals and human heart samples require further investigation to inform future clinical applications of this technology.

The increase in CV previously observed when the UnpatCPs were applied to MI hearts is an interesting and promising result.[24] Despite the observed reduction in CV for ex-vivo healthy hearts upon application of the UnpatCPs, in vivo implantation of the patch in healthy hearts showed that it did not influence the proarrhythmic state of the heart under stress. Nonetheless, from both studies it appears that an ideal case would be to tune the conductive properties of the cardiac patches so that they increase the CV in the infarct area closer to the values expected of healthy heart tissue. This could conceivably be achieved with a deeper understanding of how to manipulate cardiac electrophysiology, along with further optimization of pattern dimensions and the material’s conductive properties. Currently, the details of the molecular mechanism by which doped-conjugated polymer based materials affect cardiac electrophysiology are not fully understood and this is an interesting question worth further investigation in future studies.

### 2.6 Ex Vivo Investigation of Mechanical Integration

**Figure 5:**
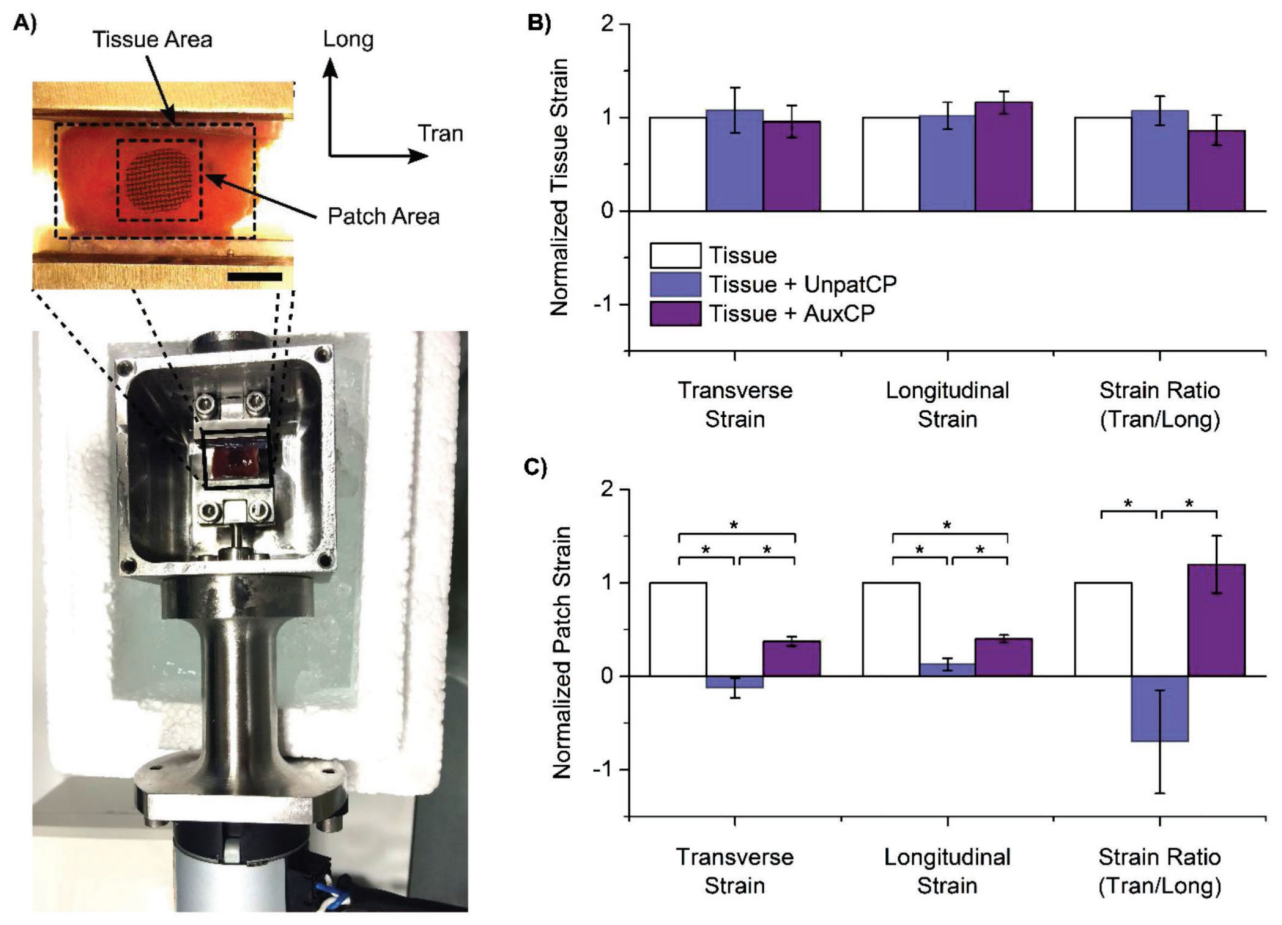
Ex vivo mechanical testing demonstrates that auxetic patches stretch and conform to the native tissue movements while providing mechanical support. A) Biomechanical rig setup with inset showing a digital optical microscope image annotated to illustrate the tissue and patch areas and the longitudinal (Long) and transverse (Tran) directions, (scale bar: 5 mm). B) Strain across the whole surrounding rat left ventricle tissue area and C) strain across the patch area for attached unpatterned (UnpatCP) or auxetic cardiac patches (AuxCP), and normalized to strain of tissue without patches. Normalized strains reported for transverse direction, longitudinal direction, and strain ratio (Tran/long) (*N* = 5, *p < 0.05; Mean ± SE).

The main purpose of micropatterning the cardiac patch was to improve its mechanical conformability to native heart tissue. In order to investigate and quantify this, a bespoke, custom-made device was built, in which dissected sections of the left ventricle could be placed into grips, and cyclically stretched at specific distances and frequencies (Figure 5A). For these experiments AuxCP-2 was used (Table S1, Supporting Information; setup number 2), as this has an anisotropy of *E*_1_/*E*_2_ = 2.79, which falls in the middle of the mechanical anisotropy range previously reported for native heart tissue (rat heart *E*_trans_/*E*_long_: 1.9 to 3.9).[27–30] The patches were always oriented to match the mechanical anisotropy of the patch to the tissue (i.e., the less stiff direction of the patch (2-direction) parallel to the less stiff longitudinal axis of the heart tissue). The patches were attached to the tissue by the previously described laser photoadhesion technique.[24]

Longitudinal strains, transverse strains, and the ratio of the two strains were measured from digital microscope videos of the tissue as it was stretched with either an AuxCP or UnpatCP attached, and normalized to the tissue before the patch was attached (Figure 5B,C). When analyzing strains across the entire tissue (≈12 × 12 mm initial tissue size) there was no significant difference in the transverse and longitudinal strains for both AuxCPs and UnpatCPs compared to the tissue without a patch (Figure 5B), suggesting that patches do not interfere with global tissue mechanics.

However, strain measurements across the patch area (≈6 × 6 mm initial patch size) demonstrated significant decreases in both longitudinal and transverse strains for UnpatCPs compared to both the tissue without a patch and with AuxCPs. Further, UnpatCPs had a normalized ratio of strains of −0.70 ± 0.6 (Tran/Long), demonstrating a dramatic reversal in mechanics. We observed a greater conformability of the AuxCPs to native tissue movement compared to the UnpatCPs. The AuxCPs significantly decreased strains across the patch area compared to the tissue without a patch. However, interestingly, although strains were reduced with the AuxCPs, they maintained a similar normalized ratio of strains as tissue without a patch (1.20 ± 0.3 and 1 ± 0, respectively), suggesting that AuxCPs stretch and conform to the native tissue movements while providing mechanical support (Figure 5C).

### 2.7 In Vivo Investigation of Cardiac Function after Myocardial Infarction

We used a rat MI model to investigate the effect of the AuxCPs on cardiac function. Once again, we used AuxCP-2 oriented to match the mechanical anisotropy of the patch to the hearts anisotropy (Figure 1B). The AuxCP was attached to the heart by the previously described laser photoadhesion technique,[24] immediately after induction of MI through permanent ligation of the left anterior descending coronary artery (LAD) (MI AuxCP, *N* = 6). We used this model as an initial proof of principle study, although a more clinically relevant scenario would be to apply the patch after the MI has resolved and scar has formed. For animal welfare reasons a second thoracotomy, which would be required for such experiments, was avoided at this stage. However, a more clinically relevant chronic heart failure model will be important in future experiments. While some progress has been made in this field to develop a less invasive surgical technique for the application of a cardiac patch to the heart after an MI,[28] currently this would still require a second thoracotomy, which is highly challenging in rodents.

**Figure 6:**
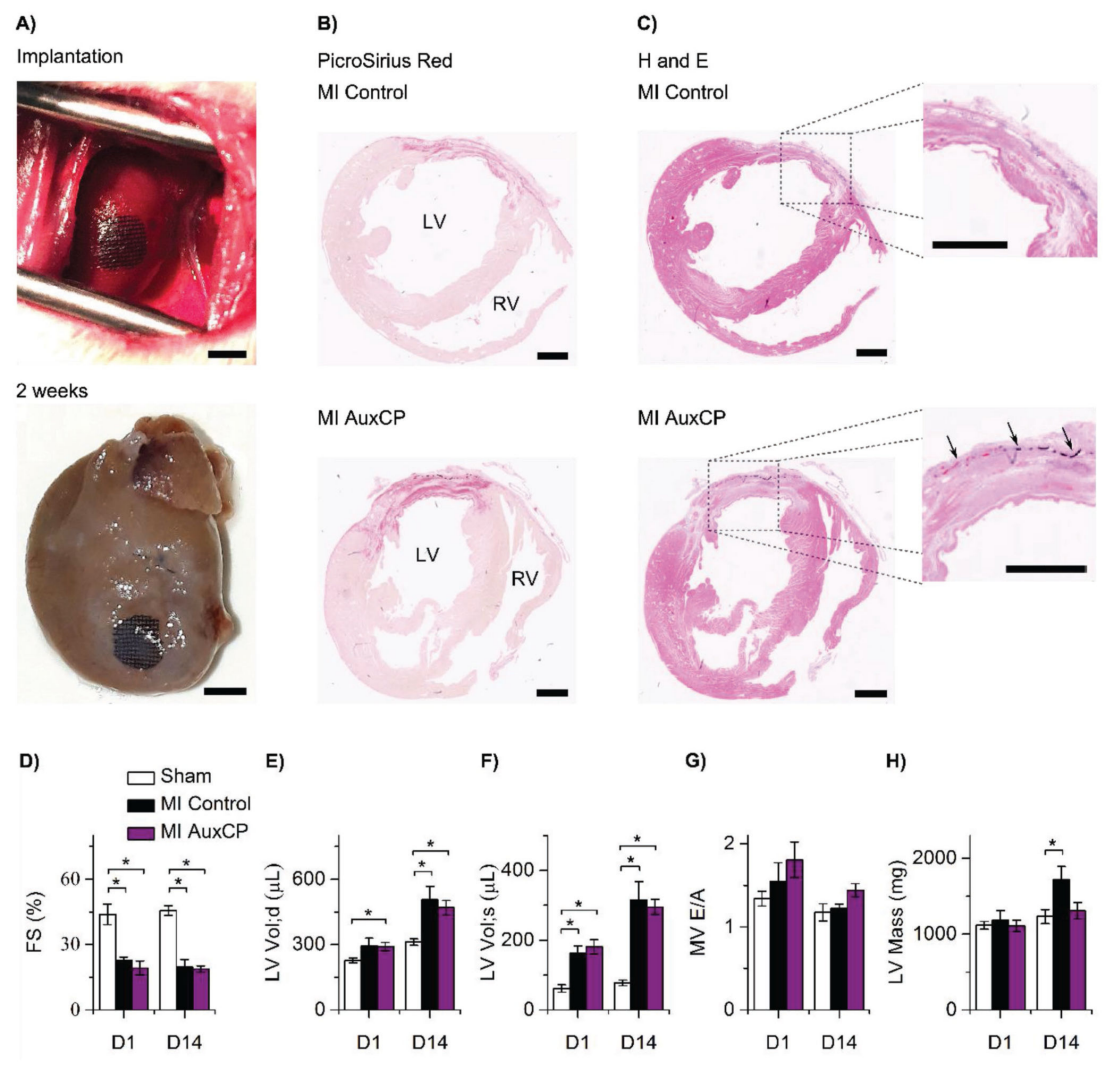
A) Representative images of the auxetic cardiac patch (AuxCP) attached via laser photoadhesion to the rat left ventricle during surgery, following a myocardial infarction (MI; permanent ligation of left anterior descending coronary artery) and a representative image of the AuxCP still attached and intact two weeks after surgery (MI AuxCP; scale bar: 4 mm). Representative histological cross-sections of an MI control and an MI AuxCP two weeks after surgery, stained with B) PicroSirius red and C) hematoxylin and eosin (H&E), arrows indicate the location of the AuxCP (scale bar: 2 mm). Ultrasound imaging for the measurement of cardiac function at day 1 and 14 after surgery, D) fractional shortening, E) LV volume at end-diastole, F) LV volume at end-systole, G) mitral valve (MV) E/A peak ratio, and H) left ventricular (LV) mass. For MI control (*N* = 4), MI AuxCP (*N* = 6) and sham (*N* = 6) hearts (*p < 0.05; Mean ± SE).

The AuxCPs remained intact and attached to the hearts two weeks after the surgeries (Figure 6A). A control group also underwent the same surgery, with an induced MI and application of the laser, but without an AuxCP applied (MI control, *N* = 4). In addition, a healthy control group underwent the same surgery, with the application of the laser, but without inducing an MI and without applying the AuxCP (sham, *N* = 6).

Histological analysis shows no significant difference between the MI controls and the MI AuxCP hearts, suggesting negligible fibrotic response to the cardiac biomaterial two weeks after the surgeries (Figure 6B,C).

Cardiac structure and function was assessed in vivo at 1 and 14 d after the surgery using ultrasound. The MI control and MI AuxCP groups showed reduced cardiac function compared to the healthy sham controls as expected. Both MI groups show similar cardiac function at 1 and 14 d after the surgeries for fractional shortening, left ventricular (LV) volume at end-diastole, LV volume at end-systole and mitral valve (MV) *E*/*A* peak ratio, suggesting that the AuxCPs have no detrimental effect on cardiac function (Figure 6D–G). However, by ultrasound measurements we also show that while the LV mass is similar for all groups at 1 day, by 14 d a significant increase in LV mass is seen for the MI control group which is not seen for the MI AuxCP group. A possible explanation may be that the patch reduces wall stress and attenuates hypertrophy (Figure 6H), as has been shown previously with other cardiac patches.[36] However, further evaluation of cardiomyocyte size and gene expression would be required to elucidate the role of the AuxCPs in attenuating hypertrophy. Greater benefit to cardiac function may have been observed over a longer follow-up period or under a chronic heart failure model, giving more time for LV remodeling to occur, which will be important considerations in future in vivo experiments for these AuxCPs.

Nevertheless, these results suggest that the AuxCPs integrate well with native heart tissue in vivo and have no detrimental effect on cardiac function, nor do they induce a significant fibrotic response. This approach to biomaterial design creates an ideal platform for future incorporation of therapeutic molecules for delivery to the epicardium.

## 3 Conclusion

We can create auxetic micropatterned cardiac patches by excimer laser microablation with mechanical properties tuned to match those of native heart tissue while maintaining the bulk properties of the material. We showed that the AuxCPs are conductive (≈10^−2^ S cm^−1^) and cytocompatible with cardiomyocytes. Ex vivo experiments demonstrated that the AuxCPs have no detrimental effect on the cardiac electrophysiology of both healthy and MI hearts. Further new ex vivo experiments showed that the AuxCPs stretch and conform to match the movements of native heart tissue, unlike the UnpatCPs. Finally, the AuxCPs integrated with native heart tissue without detrimental effect on cardiac function in a rat MI model over two weeks in vivo.

This study demonstrates a conductive cardiac patch improved through the use of an auxetic design, which can be tuned to match the mechanical demands of the heart and is a promising cardiac patch. Further, there are still many unanswered questions in the cardiac field as to the appropriate mechanics and conductivity of patches and our auxetic patch design is a promising tool for investigating optimal properties due to its high degree of tunability. Hence, the findings presented here may lead to improved biomaterial designs toward treating myocardial infarction.

## 4 Experimental Section

Extended experimental descriptions can be found in the Supporting Information.

All chemical reagents were purchased from Sigma Aldrich (UK) or VWR (UK) unless specifically noted.

All animal procedures were carried out in accordance with the UK Home Office Animals (Scientific Procedures) Act 1986 and Directive 2010/63/EU of the European Parliament on the protection of animals used for scientific purposes.

### Fabrication of Auxetic Cardiac Patches—Fabrication and Physical Characterization of Chitosan-Polyaniline Films

The chitosan-polyaniline films were fabricated by the method previously described by Mawad et al.[24] For the AuxCPs, chitosan films were micropatterned with a re-entrant honeycomb (bow-tie) pattern, before coating with polyaniline and phytic acid.

### Fabrication of Auxetic Cardiac Patches—Micropatterning by Excimer Laser Microablation

Chitosan films on glass slides were placed on the XYZ stage (accuracy ± 1 μm) of a custom-built excimer laser processing system with an LPX305i Excimer Laser (Lambda-Physik) charged with krypton fluoride gas (emitting 25 ns pulses of light at 248 nm). The light pulses passed through various optics in order to illuminate the part of the sample to be ablated, as described previously.[37] The metal mask (0.15 mm thick brass) used in this work contained the enlarged (by five times) re-entrant honeycomb geometry (12.5 × 12.5 mm^2^), which was then focused on the sample through a 5× projection lens (2.5 × 2.5 mm^2^). The programmable XYZ sample stage moved the sample by a precise distance for the ablation to be repeated; hence, fabrication of larger patch areas of a repeating pattern was possible. The optimum conditions for ablating the chitosan films were identified as 600 pulses at 350 mJ cm^−2^ delivered at 40 Hz.

### Characterization of Tunable Mechanical Properties—Mechanical Characterization

Tensile tests were carried out on patches with 6 × 6 repeat units in both 1- and 2-directions of the patch (directions annotated in Figure 1A). Precise length and width measurements were known from the fabrication technique. Thickness was determined by scanning electron microscopy, operated at 10 kV (Figure S6, Supporting Information, 29.12 ± 6.8 μm, *N* = 3, *n* = 5). Patches were wetted with deionized water and mounted using custom made stainless steel grips on an Electroforce 3200 mechanical tester with a 250 g load cell (TA Instruments, New Castle, DE) controlled by WinTest software (Ver. 7). Patches were strained to failure at a rate of 0.1% strain s^−1^, assuming quasi-static loading. The effective stiffnesses (*E*) were determined by taking the slope of a regression within the initial linear region of the stress–strain curve up to 10% strain. The anisotropic ratio of effective stiffnesses (*E*_1_/*E*_2_) was calculated by dividing the mean of *E*_1_ (*N* = 10) by the mean of *E*_2_ (*N* = 10). UTS was measured as the maximum stress reached and the strain-at-failure was taken as the strain at the UTS point.

### Ex Vivo Investigation of Cardiac Electrophysiology—Myocardial Slice Measurements

Live rat left ventricular myocardial slices were prepared according to the protocol described by Watson et al.[38] Myocardial slice force transducer and microelectrode array measurements were recorded as previously described by Mawad et al.[24]

### Ex Vivo Investigation of Cardiac Electrophysiology—MI Surgical Procedure and Ex Vivo Optical Mapping Experiments

Adult male S-D rats (250–350 g) were subjected to MI induced by ligation of the LAD and optical mapping was performed as described previously.[24] Data were analyzed using a bespoke MATLAB script as described previously.[24,39]

### Ex Vivo Investigation of Mechanical Integration

A custom-built device was used to cyclically load rat left ventricles with and without an AuxCP or UnpatCP attached. The heart was rapidly explanted and rinsed free of blood in ice-cold oxygenated Krebs–Henseleit solution (composition in mM: 119 NaCl, 4.7 KCl, 0.94 MgSO_4_, 1 CaCl_2_, 1.2 KH_2_PO_4_, 25 NaHCO_3_, 11.5 glucose, and equilibrated with 95% O_2_ + 5% CO_2_), containing heparin (12 U mL^−1^) and 30 × 10^−3^ m 2,3-butanedione monoxime (BDM). BDM is a myosin ATPase inhibitor used to inhibit contraction and reduce tissue damage during dissection and cyclic stretching. The right ventricle was removed, and the septum bisected to open the left ventricle. The papillary muscles were cut, and remaining septum removed to leave the left ventricular free wall intact and open flat. The apical and basal ends of the left ventricular free wall were glued via the epicardial surface onto a plastic insert for the biomechanical rig.

The biomechanical rig device consists of a linear actuator which can be set to cycle at specific distances, rates, and frequencies. The tissue was marked with ink in a grid formation, for image analysis. The tissue was cycled ±1 mm under tension at 1 Hz and filmed with a Dino-lite Edge AM4815ZT digital microscope (Brunel Microscopes Ltd., Wiltshire, UK). The AuxCP or UnpatCP (≈10 × 10 mm) was then secured to the epicardium using the laser technology described and shown to be safe for tissue applications previously.[24] Briefly, the polyaniline free border of the patch (1–2 mm), containing chitosan and Rose Bengal (0.1 w/v%), was irradiated by a green diode-pumped solid state laser (532 nm; CNI Lasers, China). The laser is set with a power of 170 mW in a continuous wave with a beam spot diameter on the tissue of 6 mm. The patch border was spot-irradiated for a total time of 4 min. The AuxCPs were always oriented so the less stiff 2-direction was aligned with the fibers. The cyclic loading was then repeated. Image processing software Fiji (free download under https://fiji.sc/) was used to calculate transverse and longitudinal strains. The strain values were then normalized to the corresponding tissue section before the patch was applied.

### In Vivo Investigation of Cardiac Function after Myocardial Infarction—MI Surgical Procedure and Patch Implantation

Adult male S-D rats (250–350 g) were subjected to MI induced by ligation of the LAD, see the Supporting Information for further details.

The patches were secured in place using laser technology described above.[24] COVA + CARD (Biom’up, France), a cardiac postoperative adhesion preventing membrane, was applied to the outside of the AuxCP (or on the epicardium in the case of the control groups), and sutured in 3–4 places, to the pericardium and thymus.

### Statistical Analysis

Mechanical properties and conductivity measurements are expressed as mean ± standard deviation (SD). In vitro, ex vivo, and in vivo measurements are expressed as mean ± standard error of the mean (SE). The nonparametric Mann–Whitney post hoc test was used for comparison (OriginPro 9.1, OriginLab Corporation). Values of *p* < 0.05 were considered significant.

## Supplementary Material

Supporting Information is available from the Wiley Online Library or from the author.

Supporting information
