# Supplementary material for: Auxetic Cardiac Patches with Tunable Mechanical and Conductive Properties toward Treating Myocardial Infarction
Source: Adv Funct Mater. Author manuscript; Available in PMC 2018 Jun 4. (PMC5985945; doi:10.1002/adfm.201800618)
Supplement: Supporting information [file NIHMS77154-supplement-Supporting_information.docx]

Copyright WILEY-VCH Verlag GmbH & Co. KGaA, 69469 Weinheim, Germany, 2016.

Supporting Information

Auxetic Cardiac Patches with Tunable Mechanical and Conductive Properties for Treating Myocardial Infarction

Michaella Kapnisi, Catherine Mansfield, Camille Marijon, Anne Geraldine Guex, Filippo Perbellini, Ifigeneia Bardi, Eleanor J Humphrey, Jennifer L Puetzer, Damia Mawad, Demosthenes C Koutsogeorgis, Daniel J Stuckey, Cesare M Terracciano, Sian E Harding, Molly M Stevens^*^

**Table S1:** Bow-tie dimensions and mechanical properties

| **Set-up No.** | | **1** | **2** | **3** | **4** | **5** | **6** | **7** | **8** | **9** |
| --- | --- | --- | --- | --- | --- | --- | --- | --- | --- | --- |
| **Bow-tie dimensions** | **A /µm** | 420 | 420 | 420 | 420 | 480 | 420 | 320 | 420 | 420 |
|  | **B /µm** | 360 | 280 | 240 | 280 | 280 | 240 | 280 | 240 | 280 |
|  | **A:B** | 1.17 | 1.50 | 1.75 | 1.50 | 1.71 | 1.75 | 1.14 | 1.75 | 1.50 |
|  | **θ /^o^** | 60 | 60 | 60 | 60 | 60 | 40 | 60 | 80 | 60 |
|  | **R /µm** | 100 | 100 | 100 | 50 | 100 | 100 | 100 | 100 | 150 |
| **Mechanical Properties** | **E_1_ /MPa** | 1.13 ± 0.2 | 1.43 ± 0.3 | 1.39 ± 0.3 | 0.39 ± 0.2 | 1.34 ± 0.2 | 1.01 ± 0.1 | 2.23 ± 0.2 | 2.57 ± 0.4 | 2.77 ± 0.5 |
|  | **E_2_ /MPa** | 0.23 ± 0.1 | 0.51 ± 0.2 | 0.78 ± 0.2 | 0.14 ± 0.04 | 0.68 ± 0.1 | 1.19 ± 0.2 | 0.40 ± 0.1 | 0.74 ± 0.1 | 1.10 ± 0.1 |
|  | **E_1_/E_2_** | 5.0 | 2.8 | 1.8 | 2.7 | 2.0 | 0.8 | 5.6 | 3.5 | 2.5 |
|  | **ν_12_** | -0.87 ± 0.3 | -0.47 ± 0.2 | -0.32 ± 0.1 | -0.51 ± 0.3 | -0.21 ± 0.1 | -0.45 ± 0.2 | -0.32 ± 0.2 | 0.00 ± 0.00 | -0.46 ± 0.3 |
|  | **ν_21_** | -0.34 ± 0.1 | -0.43 ± 0.3 | -0.50 ± 0.2 | -0.38 ± 0.2 | -0.84 ± 0.4 | -1.45 ± 0.2 | -0.18 ± 0.1 | 0.08 ± 0.1 | -0.84 ± 0.3 |
|  | **UTS_1_ /MPa** | 1.58 ± 0.9 | 1.15 ± 0.6 | 1.12 ± 0.6 | 0.06 ± 0.03 | 0.42 ± 0.2 | 1.12 ± 0.5 | 0.70 ± 0.4 | 0.24 ± 0.2 | 1.49 ± 0.6 |
|  | **UTS_2_ /MPa** | 0.60 ± 0.3 | 0.72 ± 0.4 | 1.46 ± 0.5 | 0.11 ± 0.03 | 0.73 ± 0.2 | 1.17 ± 0.6 | 0.76 ± 0.4 | 0.61 ± 0.4 | 0.70 ± 0.2 |
|  | **ε_1_ /%** | 47 ± 25 | 27 ± 6 | 34 ± 10 | 27 ± 12 | 34 ± 12 | 55 ± 7 | 49 ± 23 | 36 ± 22 | 40 ± 8 |
|  | **ε_2_ /%** | 55 ± 25 | 37 ± 8 | 28 ± 4 | 50 ± 26 | 44 ± 10 | 50 ± 15 | 96 ± 29 | 40 ± 12 | 48 ± 14 |

**
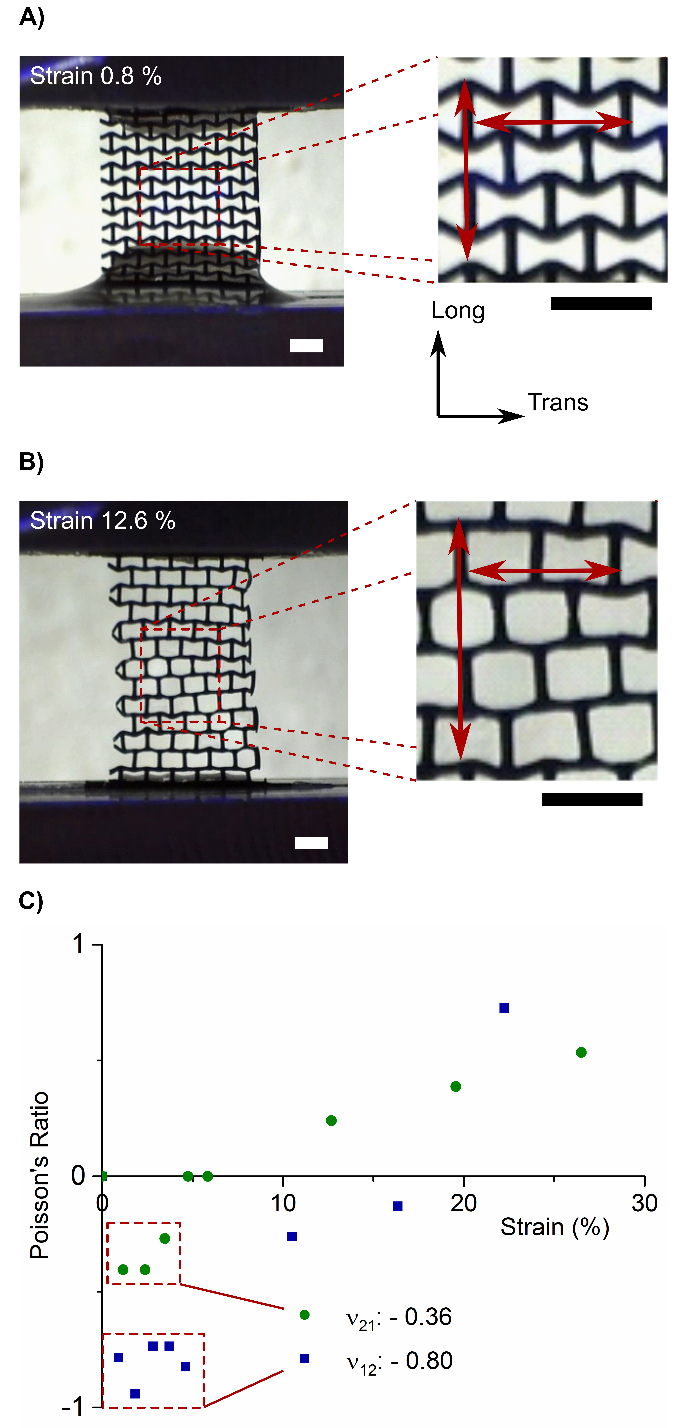
**

**Figure S1 A and B)** Digital optical microscope images of the AuxCPs during tensile testing at **A)** 0.8 % strain and **B)** 12.6 % strain (scale bars: 1 mm), including annotation to indicate the measurements taken to calculate the Poisson’s ratio. **C)** Representative graph of Poisson’s ratio (in each direction; ν_12_ and ν_21_) against strain.

**
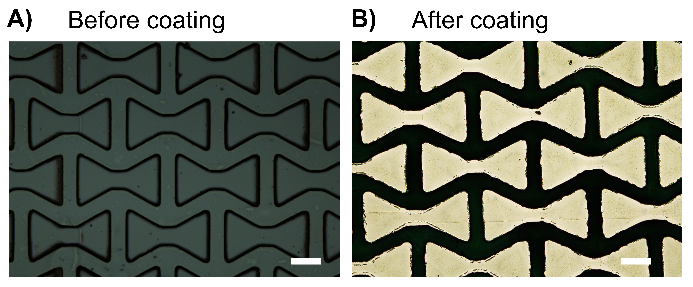
**

**Figure S2 A and B)** Optical microscope images of the AuxCPs created by excimer laser microablation **A)** before and **B)** after coating with polyaniline and phytic acid (scale bars: 200 µm).


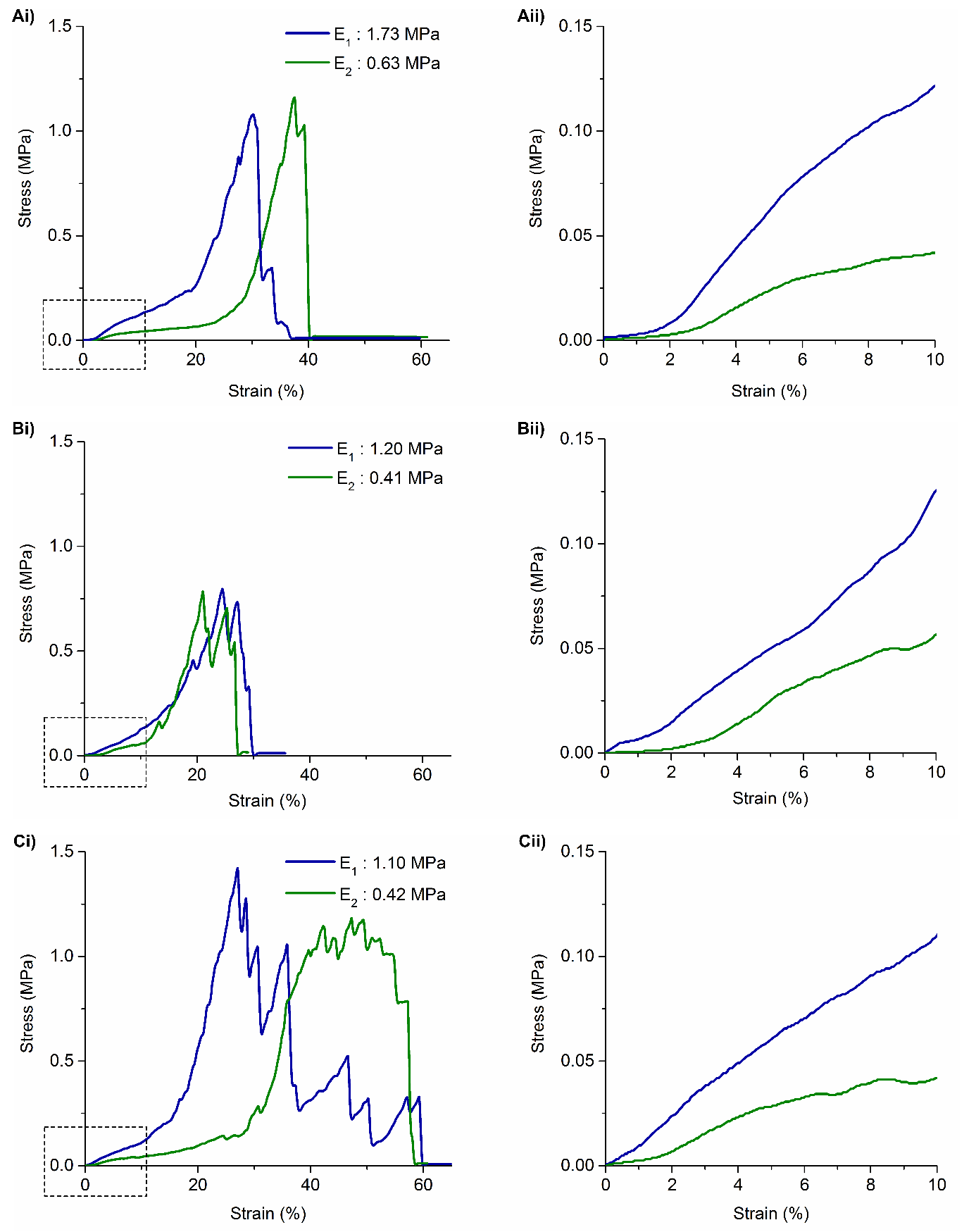


**Figure S3:** **A-C)** Three separate sets of representative stress-strain curves for an auxetic cardiac patch (AuxCP, set-up 2) in both the 1 and 2-directions, showing **i)** the whole tensile test and **ii)** the first 10 % strain only.


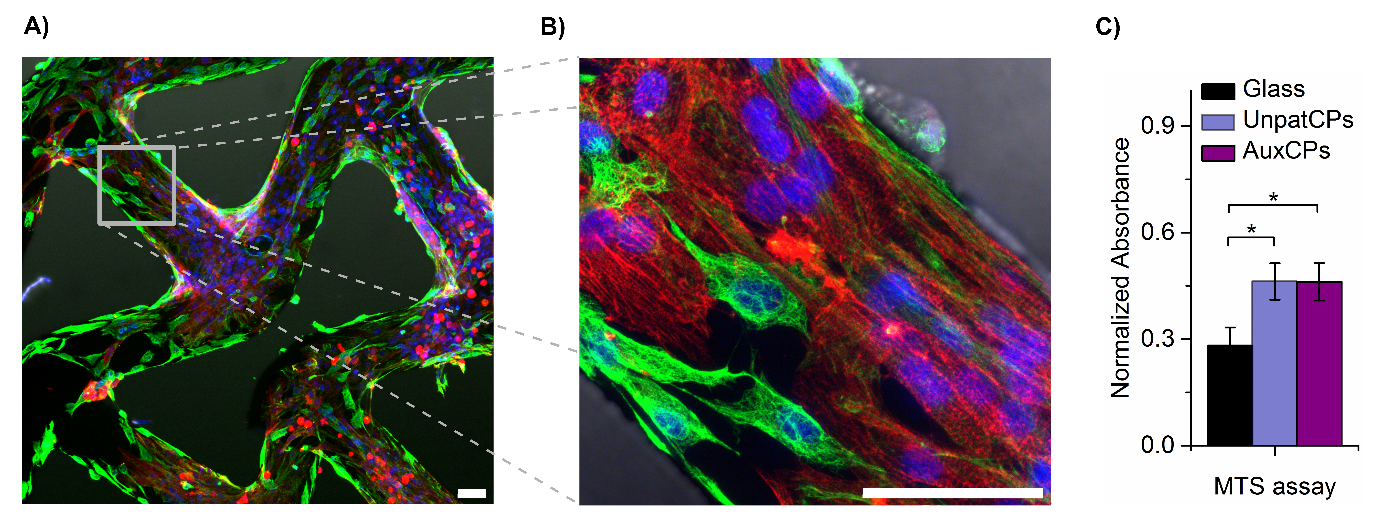


**Figure S4: A and B)** Confocal microscopy images of neonatal rat ventricular myocytes and fibroblasts cultured on the auxetic cardiac patches (AuxCPs) for 3 days, fluorescently stained for vimentin (green), α-actinin (red) and nuclei (blue), (scale bar: 50 µm.) **C)** Cell metabolic activity determined by an MTS assay of neonatal rat ventricular myocytes and fibroblasts cultured on glass controls, unpatterned cardiac patches (UnpatCPs) and AuxCPs after 3 days in culture, (N = 6, **p* < 0.05; Mean ± SE.)

**Table S2:** List of bow-tie dimensions and mechanical properties for AuxCP-10, discussed in section 2.5.2.

| **Bow-Tie Dimensions** | | | | | **Mechanical Properties** | | |
| --- | --- | --- | --- | --- | --- | --- | --- |
| **A /µm** | **B /µm** | **A:B** | **θ /^o^** | **R /µm** | **E_1_ /MPa** | **E_2_ /MPa** | **E_1_/E_2_** |
| 425 | 245 | 1.73 | 36 | 100 | 2.16 ± 0.2 | 1.33 ± 0.1 | 1.63 ± 0.2 |


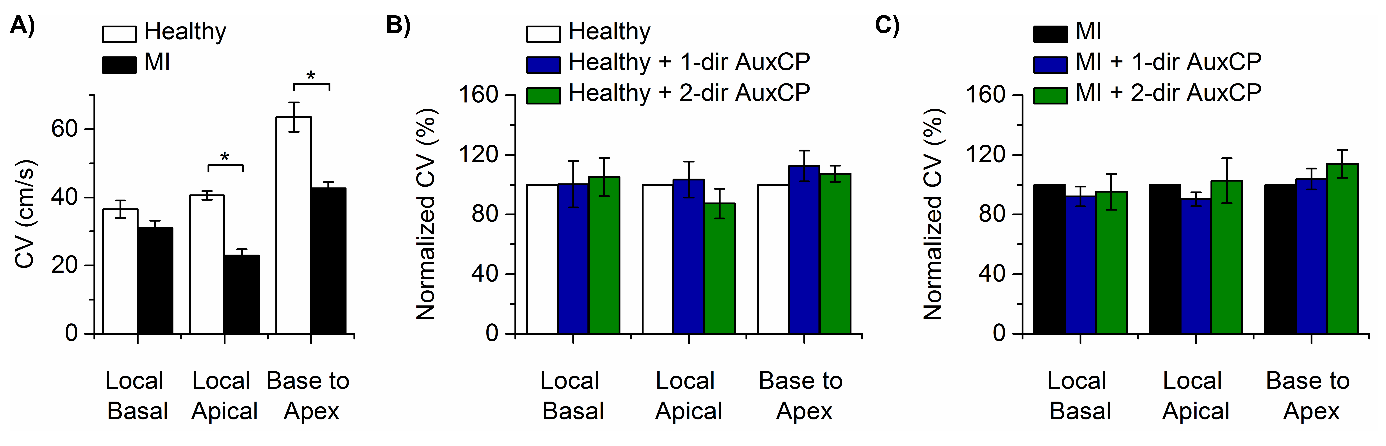


**Figure S5:** Optical mapping measurements on whole hearts with AuxCP-10 attached. **A)** Conduction velocity (CV) in healthy and MI hearts, (N = 10, **p* < 0.05; Mean ± SE). CV in **B)** healthy hearts and **C)** MI hearts after patch application, normalized to the hearts without the patch, (N = 4-5; Mean ± SE).


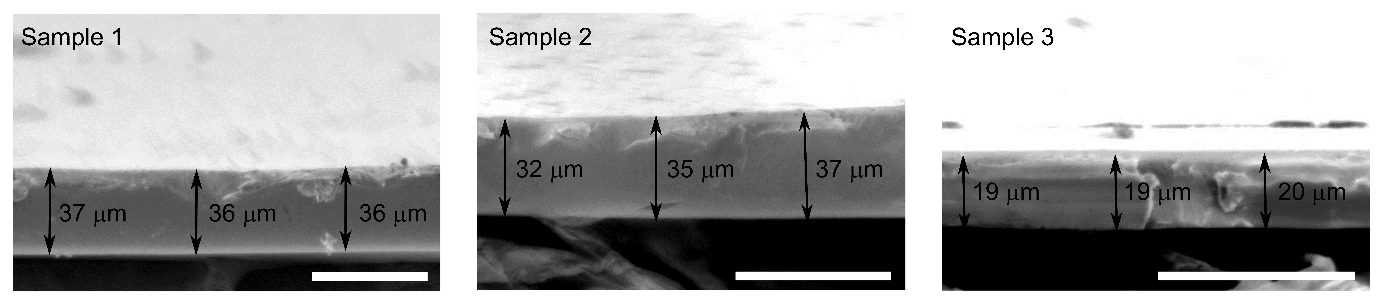


**Figure S6:** Representative scanning electron microscopy (SEM) images of 3 different samples of the chitosan-polyaniline thin film patches (UnpatCPs) to illustrate how the film thickness was calculated. Film thickness = 29.12 ± 6.8 µm. (scale bar = 50 µm; N = 3, n = 5, Mean ± SD).

**Extended Experimental Section**

**Characterization of the Poisson’s Ratio**

The Poisson’s ratio (ν) is the negative ratio of the transverse strain (ε_T_) to longitudinal strain (ε_L_). Poisson’s ratio measurements were calculated from Dino-lite Edge AM4815ZT digital microscope videos (Brunel Microscopes Ltd, Wiltshire, UK) of the tensile tests. The images were processed using a method similar to that previously described by Clausen *et al.*^[17]^ The MATLAB Image Processing Toolbox was used to convert the videos into still images and then contour plots. Image processing software Fiji was used to measure all distances in pixels across the length and width of the central 2 x 2 repeat units to calculate the transverse (ε_T_) and longitudinal strain (ε_L_) for all images and therefore determine the Poisson’s ratio, ($\nu= - \frac{\varepsilon T}{\varepsilon L}$). **Characterization of Conductivity**

The electrodes were gold-coated Mylar (VacuLayer Corp) held in place on the conductive side of the patch and connected to an eDAQ EA163 potentiostat. The *I-V* characteristics of the films were recorded by sweeping the voltage between ± 1 V at 100 mV·s^­1^. The resistivity (*ρ*) was calculated according to **Equation (S1)**:

$\rho=\frac{A}{L}R$ (S1)

where *R* is the resistance of the film calculated from the slope of the *V* vs *I* plot, *A* is the cross-sectional area of the film (0.15 mm) and L is the distance between the two electrodes (5 mm). The conductivity (σ) could then be calculated from **Equation (S2)**:

$\sigma=\frac{1}{\rho}$ (S2)

**Neonatal Rat Ventricular Myocytes Cultured on Auxetic Cardiac Patches**

*Cell culture:* Chamber slides were coated with 1.5 w/v% sterile agarose to block cell adhesion to the bottom of the slide. Patches were placed in each well and held in place by sterile silicone o-rings (Polymax Ltd, UK). The patches were then sterilized with UV for 30 minutes and incubated with sterile water followed by FBS for 48 hours each, prior to cell seeding.

Neonatal rat ventricular myocytes (NRVMs) were isolated from Sprague-Dawley (S-D) rats 0-2 days after birth by excising the hearts and isolating the ventricles. A GentleMACs neonatal heart dissociation kit (Miltenyi Biotec) was used to enzymatically digest the ventricles to produce a cell suspension containing NRVMs and fibroblasts. Each patch was seeded with 200,000 NRVMs and fibroblasts in NRVM medium. All experiments were conducted at 2-3 days post seeding unless stated otherwise.

*MTS assay:* Cell metabolic activity was also assessed by quantification of the reduction of MTS (3-(4,5-dimethylthiazol-2-yl)-5-(3-carboxymethoxyphenyl)-2-(4-sulfophenyl)-2H-tetrazolium) (Promega) to a colored formazan product, and the absorbance was read on a Promega Glomax multi detection system at 490 nm.

*Immunofluorescence staining:* NRVMs and fibroblasts were stained for α-actinin and vimentin, respectively, with the following antibodies: mouse anti- α-actinin primary antibody (diluted 1:1000), followed by goat anti-mouse Cy3 secondary antibody (1:1000) (Millipore) and rabbit anti-vimentin primary antibody (1:1000), followed by donkey anti-rabbit Alexa 488 secondary antibody (1:1500), before mounting in Vectashield (Vectalabs) mounting medium containing DAPI. The cells were imaged using an inverted Zeiss LSM-780 confocal microscope. Image processing software Fiji was used for all image analysis.

***In vivo* investigation of cardiac function after myocardial infarction**

*Animal Preparation:* Animals were ordered from Charles River Laboratories and left to acclimatize for at least 1 week before the surgery was performed. Anesthesia was induced with 5 % isoflurane (Zoatis, UK) and surgical anesthesia was confirmed by the loss of standing and pedal reflexes. The hairs were removed from the left part of the thorax using clippers (Harvard Apparatus, UK) and the eyes were hydrated with Lacri-lube® (Allergan, UK). Buprenorphine (0.05 mg/kg, Vetergesic®, Ceva, UK) diluted in saline was administered subcutaneously for analgesia and fluid therapy. Rats were intubated with an appropriate cannula (Williams Medical Supplies, UK) and mechanically ventilated (Harvard Apparatus, UK). Ventilation parameters were adjusted based on the animal weight and chest movement. Body temperature was maintained during the whole procedure using an adjustable heating mat. The surgical site was then scrubbed using diluted iodine solution and isoflurane was then reduced and maintained at 2 % during the whole procedure.

*MI, Patch Implantation and Aftercare:* The heart was exposed by a horizontal incision of the skin followed by the dissociation of the thorax and intercostal muscles to access the base of the left ventricle. The pericardium was ruptured, and the MI was induced by ligation of the left anterior descending coronary artery (LAD), ligated 1-2mm distal to the inferior border of the left atrium using a 7/0 Prolene suture. Effective ligation was confirmed by blanching and cyanosis of the left ventricular free wall and apex. The patch was secured in place as described earlier.

The rib cage and muscles were then closed with a resorbable 4/0 suture (PDS™II, Ethicon, Belgium). The anesthesia was turned down to 1 % isoflurane and the skin was closed with the same suture. At the end of the surgery, the anesthetic gas was turned off and the animal was extubated and placed in a heat chamber until full recovery. Animals were checked regularly for signs of pain/distress and analgesia was maintained for 1 day using Buprenorphine (0.05 mg/kg, Vetergesic, Ceva, UK).

*Heart Isolation:* At the end of the experiment, the hearts were isolated under non-recovery anesthesia. Briefly, the chest of the animal was opened to expose the heart. A solution of potassium chloride (50 mM in DI-H_2_O) was injected in the left ventricle through the apex to stop the heart in diastole, followed by a solution of 3.7 % PFA to fix the tissue *in situ*. The heart was then removed, placed in 3.7 % PFA for 24 hours at room temperature, and was then transferred to 70 % ethanol before preparation for histology.

*Ultrasound Imaging for the Measurement of Systolic and Diastolic Function:* Ultrasound measurements were performed on a Vevo 2100 system (VisualSonics, California, CA, USA) using an MS550D 30 MHz transducer (VisualSonics). Rats were anaesthetized with 1.5–2.0 % isoflurane in 2 L·min^-1^ oxygen and positioned supine on a physiological monitoring platform which simultaneously regulated body temperature, whilst measuring respiration and ECG traces. Prior to imaging, hair was removed from the chest to reduce the attenuation of the ultrasound signal.

For the assessment of systolic function, parasternal long axis m-mode images were acquired and volumes were calculated using Teichholz's formula.^[S1]^ For the assessment of diastolic function, we used color B mode guided, pulsed wave, Doppler mode ultrasound to measure mitral inflow in an apical four chamber view of the heart. E and A peaks were measured from three consecutive heart beats, and the mean E/A ratio was calculated for each animal.^[S2]^ LV mass was calculated from short axis m-mode images using the standard formula as follows:

$${LV mass=((LVIDd+PWDd+AWDd)}^{3}-\mathrm{LVIDd}^{3}) x 1.05$$

Where LVIDd is the left ventricular internal dimension at diastole, PWDd is the posterior wall dimension at diastole, AWDd is the anterior wall dimension at diastole and 1.05 is the specific gravity of the myocardium.

*Histology and Immunohistochemistry:* Histology and immunohistochemistry were carried out as previously described.^[40]^ Briefly, the isolated hearts were cut into 2 mm thick cross-sections and embedded in paraffin wax. The tissue-paraffin blocks were cut into thin sections (5 µm) using a microtome and mounted onto glass slides. The slides were then stained with either PicroSirius Red or hematoxylin and eosin (H and E). The samples were examined with a wide field microscope. Image processing software Fiji (ImageJ) was used for all image analysis.

**Extended References**

[S1] M. G. Hasham, N. Baxan, D. J. Stuckey, J. Branca, B. Perkins, O. Dent, T. Duffy, T. S. Hameed, S. E. Stella, M. Bellahcene, M. D. Schneider, S. E. Harding, N. Rosenthal, S. Sattler = *Dis. Model. Mech.* **2017,** *10,* 259–270.

[S2] T. A. Roberts, A. N. Price, L. H. Jackson, V. Taylor, A. L. David, M. F, Lythgoe, D. J. Stuckey, *NMR Biomed.* **2017,** *30,* 1–10.
